# Systematic review and meta-analysis of the diagnostic accuracy of prostate-specific antigen (PSA) for the detection of prostate cancer in symptomatic patients

**DOI:** 10.1186/s12916-021-02230-y

**Published:** 2022-02-07

**Authors:** Samuel W. D. Merriel, Lucy Pocock, Emma Gilbert, Sam Creavin, Fiona M. Walter, Anne Spencer, Willie Hamilton

**Affiliations:** University of Exeter, 1.18 College House, St Luke’s Campus, Exeter, Devon UK

**Keywords:** Prostate-specific antigen, PSA, Lower urinary tract symptoms, LUTS, Prostate cancer, Diagnostic accuracy, Primary care, Secondary care

## Abstract

**Background:**

Prostate-specific antigen (PSA) is a commonly used test to detect prostate cancer. Attention has mostly focused on the use of PSA in screening asymptomatic patients, but the diagnostic accuracy of PSA for prostate cancer in patients with symptoms is less well understood.

**Methods:**

A systematic database search was conducted of Medline, EMBASE, Web of Science, and the Cochrane library. Studies reporting the diagnostic accuracy of PSA for prostate cancer in patients with symptoms were included. Two investigators independently assessed the titles and abstracts of all database search hits and full texts of potentially relevant studies against the inclusion criteria, and data extracted into a proforma. Study quality was assessed using the QUADAS-2 tool by two investigators independently. Summary estimates of diagnostic accuracy were calculated with meta-analysis using bivariate mixed effects regression.

**Results:**

Five hundred sixty-three search hits were assessed by title and abstract after de-duplication, with 75 full text papers reviewed. Nineteen studies met the inclusion criteria, 18 of which were conducted in secondary care settings with one from a screening study cohort. All studies used histology obtained by transrectal ultrasound-guided biopsy (TRUS) as a reference test; usually only for patients with elevated PSA or abnormal prostate examination. Pooled data from 14,489 patients found estimated sensitivity of PSA for prostate cancer was 0.93 (95% CI 0.88, 0.96) and specificity was 0.20 (95% CI 0.12, 0.33). The area under the hierarchical summary receiver operator characteristic curve was 0.72 (95% CI 0.68, 0.76). All studies were assessed as having a high risk of bias in at least one QUADAS-2 domain.

**Conclusions:**

Currently available evidence suggests PSA is highly sensitive but poorly specific for prostate cancer detection in symptomatic patients. However, significant limitations in study design and reference test reduces the certainty of this estimate. There is very limited evidence for the performance of PSA in primary care, the healthcare setting where most PSA testing is performed.

**Supplementary Information:**

The online version contains supplementary material available at 10.1186/s12916-021-02230-y.

## Background

Prostate-specific antigen (PSA) is a commonly used test for the detection of prostate cancer, identifying patients that may require a diagnostic test [1]. PSA testing is usually performed for one of two reasons: assessing a patient presenting to their general practitioner (GP) or primary care physician with lower urinary tract symptoms (LUTS) [2] or screening for a patient who is asymptomatic but concerned about their risk of prostate cancer [3, 4]. Patients with an elevated PSA are usually referred to a urologist for diagnostic testing, which may include magnetic resonance imaging (MRI) of the prostate and/or a prostate biopsy [5]. Very large randomised controlled trials of PSA-based prostate cancer screening have been performed; these are summarised in a recent systematic review in 2018 that showed a small potential reduction in prostate cancer specific mortality with no change in all-cause mortality and an increased risk of complications from biopsy, overdiagnosis of clinically insignificant prostate cancer, and overtreatment [6–8]. However, uncertainty remains about the diagnostic accuracy of PSA for prostate cancer in patients with LUTS [9].

The most recent systematic review of the diagnostic accuracy of PSA was published by Harvey et al. in 2009 [10]. A range of estimates for the accuracy of PSA was found amongst the ten included studies. That review presented limited information on their methods; crucially, it was unclear whether the included studies were assessing PSA in symptomatic or asymptomatic patients nor was it clear whether any were relevant to primary care populations. Just et al. published a brief review of the literature in 2018, highlighting that the paucity of research in this area applicable to primary care, where a significant proportion of PSA testing is performed, still remains [9].

This systematic review aimed to determine the diagnostic accuracy of PSA for the detection of prostate cancer in patients, focusing on studies where the included patients (or a subset of included patients) had at least one symptom that could relate to an undiagnosed prostate cancer. Given the findings by Just et al., this review considered studies from primary and secondary care settings.

## Methods

### Types of studies

We included cross-sectional and cohort studies that reported paired data on the diagnostic accuracy of PSA for the detection of prostate cancer in symptomatic men, verified with the use of a reference test (prostate biopsy). We excluded studies if it was not possible to extract data for a complete two-by-two table for the target condition or if the patient cohort was only asymptomatic patients (i.e. a screening cohort). We did not restrict studies by publication date, country, or clinical setting.

### Participants

The study population of interest was any patient with symptoms of a possible prostate cancer, with no history of the disease. We defined symptoms of prostate cancer as at least one of LUTS (nocturia, hesitancy, poor stream, incomplete voiding, double voiding, terminal dribbling, urgency, incontinence, frequency), haematuria, erectile dysfunction, or lower back pain. Symptoms may have been identified by a standardised tool, such as the International Prostate Symptom Score (IPSS), clinical coding, or through patient self-report. We did not exclude studies based on age of participants or study setting. Where studies included groups of both asymptomatic and symptomatic men, we included men in the symptomatic group.

### Index test

The index test was prostate-specific antigen (PSA) in a peripheral blood sample, measured in nanograms per millilitre (ng/mL). We did not set an a priori PSA threshold for prostate cancer detection but instead extracted data based on the PSA thresholds used in each study.

### Target condition

The target condition was prostate cancer, regardless of Gleason grade or clinicopathological stage.

### Reference test

The reference test was a biopsy of the prostate with histological examination. We did not set an inclusion criteria on the basis of prostate biopsy approach used in studies, but this was recorded as part of the data extraction.

### Electronic searches

Medline Ovid, EMBASE, the Cochrane Central Register of Controlled Trials (CENTRAL), and Web of Science databases were utilised to identify relevant studies. Key search terms, informed by the Scottish Intercollegiate Guidelines Network (SIGN) search strategies and pre-existing systematic reviews in the field of prostate cancer, were combined with MeSH terms for each database search. Hand-searching of reference lists from included studies and snowballing techniques were performed to locate any other possibly relevant studies. Please see Additional file 1 for the search strategy used in this review.

### Data collection and analysis

#### Selection of studies

Search hits from each database were downloaded and combined into a review database managed in Mendeley Desktop. Each search hit was screened against the inclusion/exclusion criteria by SM and a 2nd investigator (LP, SC, or EG) independently, based on title and abstract. Full text articles were reviewed if a reviewer was unclear on the basis of title and abstract. Any discrepancies of study inclusion were adjudicated by a third reviewer (WH or AS).

#### Data extraction

A pre-prepared proforma for data extraction was used to collate relevant data from each included study, including two by two tables for the index and reference tests. SM extracted the data from all included studies. A second investigator extracted data from a random sample of 10% of included studies for verification of accuracy of data extraction. Any discrepancies were adjudicated by a third reviewer (WH or AS).

#### Quality assessment

Risk of bias and applicability of all included studies was assessed by SM using the QUADAS-2 [11] tool, with a second investigator independently assessing 10% of included studies and discussed any discrepancies with SM.

#### Meta-analysis

Raw data extracted from included papers on PSA result and prostate cancer diagnoses were extracted and combined into 2 × 2 tables to assess diagnostic accuracy. Measures of pooled diagnostic accuracy were intended to be determined for the following outcomes using bivariate mixed effects regression [12]:

Any prostate cancer diagnosis

Clinically significant prostate cancer diagnosis (Gleason Grade Group ≥ 2)

The majority of included studies used a fixed PSA threshold of 4 ng/mL, and this was also used as the threshold for meta-analysis. No included studies reported sufficient information to Meta-analyse age-adjusted thresholds.

#### Heterogeneity

Heterogeneity was assessed for visually, using Forest plots of sensitivity and specificity.

All analyses were performed using Stata Version 16 (StataCorp, http://www.stata.com)

### Protocol publication

The protocol for this systematic review and meta-analysis was registered with PROSPERO (CRD42021257783).

### PRISMA reporting guidelines

This systematic review was conducted following the PRISMA reporting guidelines for systematic reviews and meta-analyses [13]. A completed PRISMA checklist can be found in Additional file 2.

## Results

**Fig. 1:**
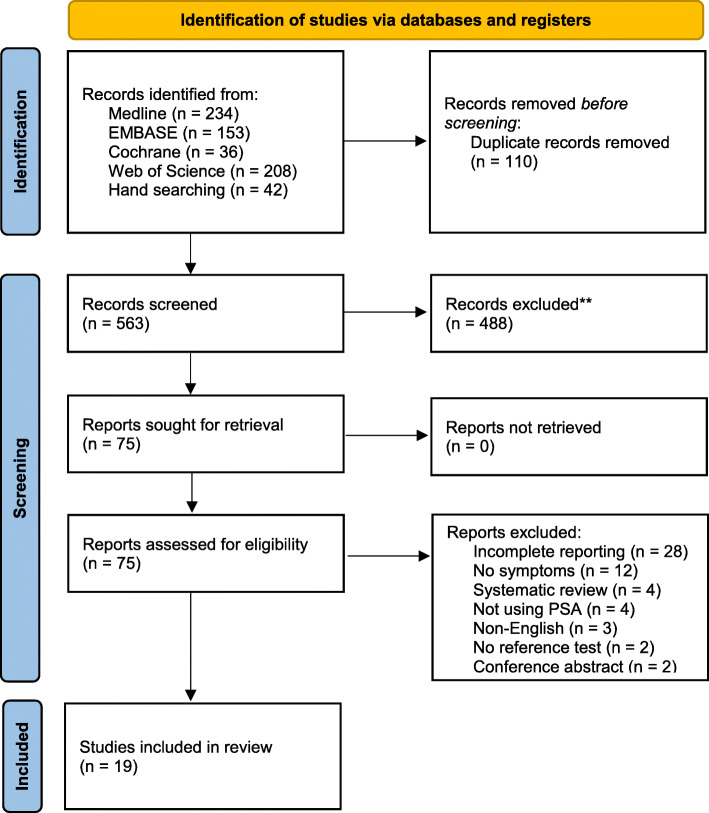
PRISMA 2020 flow diagram

Database searching identified 631 potentially relevant studies, and a further 42 studies were identified through reference list checking and snowballing techniques from initial search hits and key papers. Following de-duplication, 563 search hits were assessed by two reviewers independently, and 75 papers selected for full text assessment. Nineteen papers were ultimately included. Details of full-text exclusions can be found in Fig. 1.

**Fig. 2:**
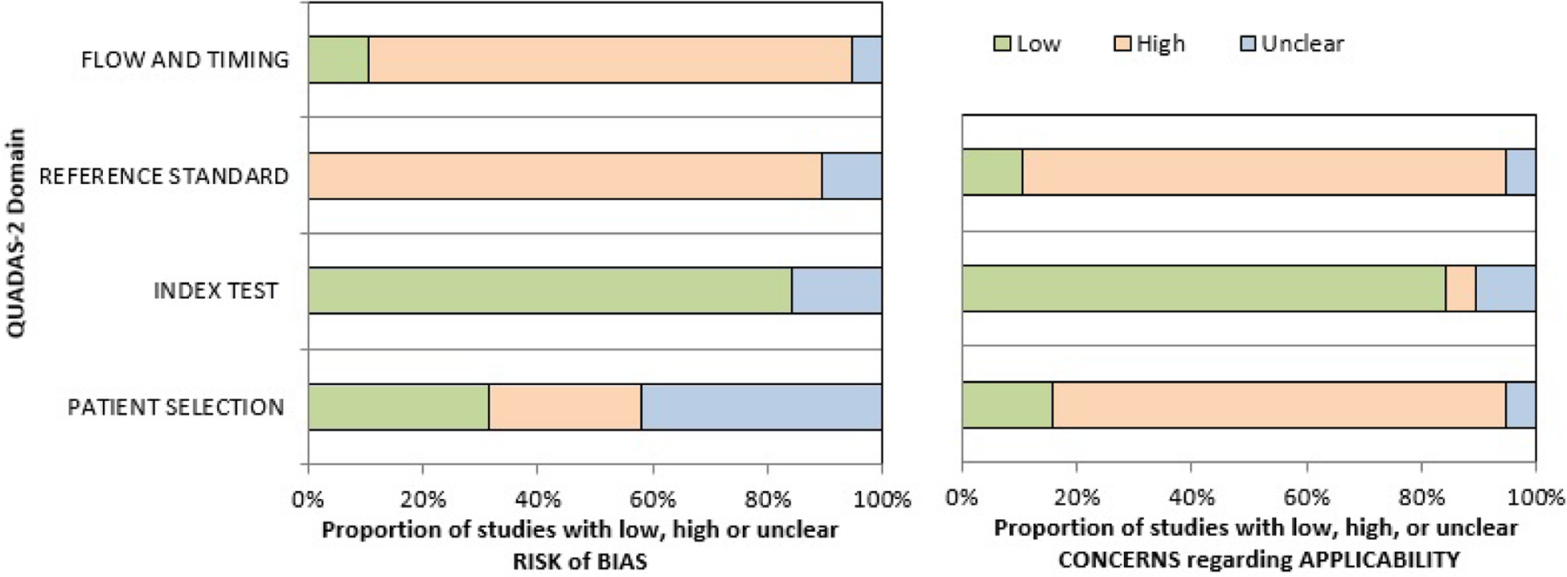
Summary of QUADAS-2 risk of bias assessments

Risk of bias assessment using the QUADAS-2 tool demonstrated a number of potential areas of bias in the included studies (see Table 1 and Fig. 2). None of the studies were assessed as having a low risk of bias with regards to the reference standard test, which was almost always a transrectal ultrasound-guided (TRUS) biopsy. TRUS biopsy suffers from a significant risk of false negative or misclassification of prostate cancer diagnosis owing to the random nature of sampling of the prostate [14]. The reference standard was performed with knowledge of the index test (PSA) in 16 of 19 studies. Patient populations were drawn from hospital urology clinics in all but one study, affecting applicability to other clinical settings. Limited information with regards to patient selection was available in eight studies, and the majority had a low risk of bias with regards to the conduct of the index test.

**Table 1:**
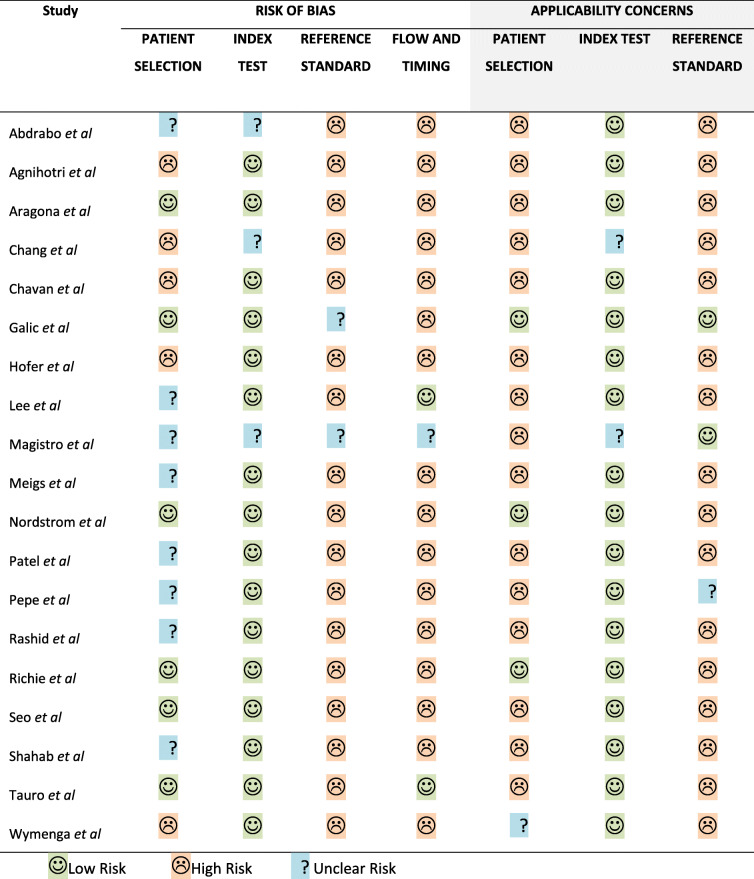
Risk of bias assessment of included studies using QUADAS-2 tool

Table 2 summarises the features of the included studies. There was a wide range of countries and study sizes. One study focused on a symptomatic cohort within a population screening study, and the remainder were set in hospital urology clinics. No study was performed in a primary care population. Five studies gathered stage and grade data. All but one study used TRUS biopsy as a reference test, with three studies also gathering diagnostic data from transurethral resection of the prostate (TURP) or other urological surgical procedures involving the prostate.

**Table 2:** Details of included studies

First author	Year	Country	Number of patients	Mean age (range)*	Setting	PSA range	Stage/grade data	Reference test
Abdrabo et al. [15]	2011	Sudan	118	70 years (56–83)	One hospital urology clinic	2.5–10 ng/mL	No	TRUS biopsy
Agnihotri et al. [16]	2014	India	875 biopsied (of 4702 patients)	66 years (50–75)	One hospital urology clinic	Any	No	TRUS biopsy
Aragona et al. [17]	2005	Italy	3171 biopsied (of 16,298 patients)	62 years (40–75)	15 hospital urology clinics	Any	Clinical TNM staging	TRUS biopsy
Chang et al. [18]	2015	Taiwan	225	PCa 72 years; BPH 67 years	One hospital urology clinic	Any	TNM stage and Gleason Score	TRUS biopsy
Chavan et al. [19]	2009	India	440 biopsied (of 922 patients)	64 years (40–95)	One tertiary hospital urology clinic	Any	No	TRUS biopsy
Galic et al. [20]	2003	Croatia	88 biopsied (of 944 patients)	≥ 50 years	Recruited from two villages to attend hospital clinic	Not stated	No	TRUS biopsy
Hofer et al. [21]	2000	Germany	188	PCa 70 years; BPH 68 years	One hospital urology clinic	Any	No	TRUS biopsy/TURP/non-cancer surgery
Lee et al. [22]	2006	Korea	201	63 years	One hospital urology clinic	< 4 ng/mL	No	TRUS biopsy
Magistro et al. [23]	2020	Germany	1125	70 years	One hospital urology clinic	Any	TNM stage and Gleason Score	HoLEP (+ mpMRI with targeted and systemic biopsy for some patients)
Meigs et al. [24]	1996	USA	1524	50-79 years	One hospital urology clinic + two BPH study cohorts	Any	Clinical T stage	TRUS biopsy/TURP/non-cancer surgery
Nordstrom et al. [25]	2021	Sweden	1554	64 years (50–69)	Population-based screening study cohort	> 3 ng/mL	TNM stage and Gleason Score	TRUS biopsy
Patel et al. [26]	2009	UK	647 biopsied (of 3976 patients)	65 years (15–91)	One hospital urology clinic	Any	No	TRUS biopsy
Pepe et al. [27]	2007	Italy	403 biopsied (of 13,294 patients)	62 years (40–75)	Two hospital urology clinics	< 4 ng/mL	Pathological T stage	TRUS biopsy
Rashid et al. [28]	2012	Bangladesh	206	> 50 years	One hospital urology clinic and one nursing home	> 2.5 ng/mL	No	TRUS biopsy
Richie et al. [29]	1993	USA	1167 biopsied (of 6630 patients)	63 years (50–96)	Six medical centres	Any	TNM stage and Gleason Score	TRUS biopsy
Seo et al. [30]	2007	Korea	4967	66 years (40–96)	25 hospital urology clinics	Any	No	TRUS biopsy
Shahab et al. [31]	2013	Indonesia	404	64 years (34–84)	One hospital urology clinic	Any	TNM stage and Gleason Score	TRUS biopsy
Tauro et al. [32]	2009	India	100	68 years	One hospital urology clinic	Any	No	TRUS biopsy
Wymenga et al. [33]	2000	The Netherlands	716	Not reported	Two hospital urology clinics	Any	Clinical T stage	TRUS biopsy/TURP/prostatectomy

*PSA* prostate-specific antigen, *TRUS* transrectal ultrasound-guided biopsy, *PCa* prostate cancer, *BPH* benign prostatic hypertrophy, *TNM* tumour-node-metastasis, *TURP* transurethral resection of the prostate, *HoLEP* holmium laser enucleation of the prostate, *mpMRI* multiparametric magnetic resonance imaging

*Age range and/or mean not present in table if not reported

**Fig. 3:**
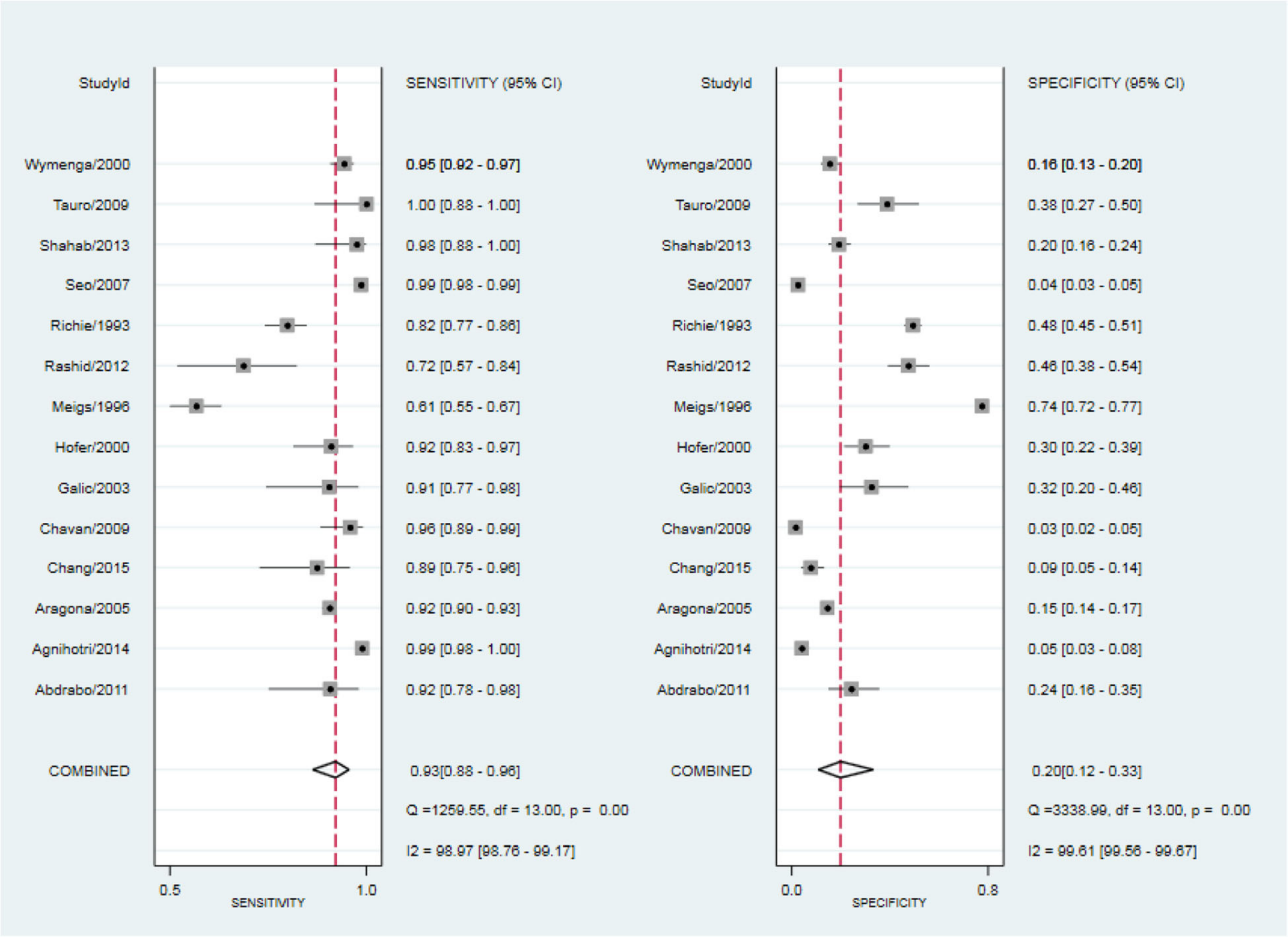
Forest plot of included studies using PSA cut-off of 4 ng/mL

**Fig. 4:**
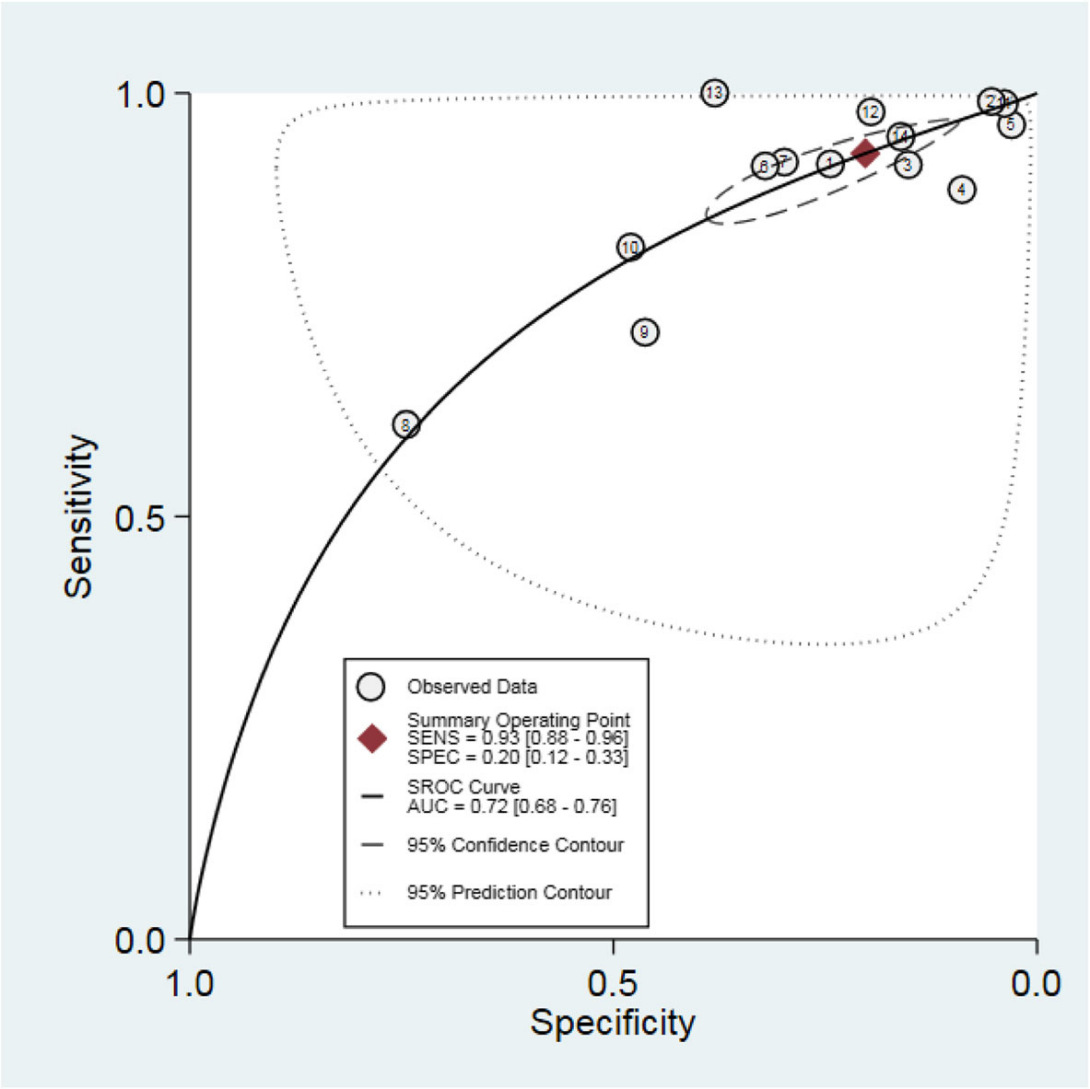
Hierarchical summary receiver operator curve (HSROC) of included studies using PSA cut-off of 4 ng/mL

Table 3 shows the measures of diagnostic accuracy calculated using reported data in 14 included studies featuring 14,489 patients that considered a PSA level of greater than or equal to 4 ng/mL as abnormal. The remaining five studies focused on populations in a specific part of the PSA range; either a low or raised PSA level. Meta-analysis showed an estimated combined sensitivity of a PSA greater than or equal to 4 ng/mL for any prostate cancer of 0.93 (95% CI 0.88, 0.96) and a combined specificity of 0.20 (95% CI 0.12, 0.33) (see Fig. 3). There was significant heterogeneity between included studies (sensitivity *I*^2^ 98.97, specificity *I*^2^ 99.61). Hierarchical summary receiver operator curve (HSROC) analysis showed an AUC of 0.72 (95% CI 0.68, 0.76) (see Fig. 4). A Fagan plot can be found in Additional File 3.

**Table 3:** Diagnostic accuracy of PSA ≥ 4 ng/mL for prostate cancer detection in symptomatic patients

Author	Year	Sensitivity	Specificity	Positive predictive value	Negative predictive value
Abdrabo	2011	0.92	0.24	0.35	0.87
Agnihotri	2014	0.99	0.05	0.59	0.80
Aragona	2005	0.92	0.15	0.38	0.76
Chang	2015	0.89	0.09	0.19	0.76
Chavan	2009	0.96	0.03	0.18	0.79
Galic	2003	0.91	0.32	0.47	0.85
Hofer	2000	0.92	0.29	0.46	0.85
Meigs	1996	0.61	0.74	0.34	0.89
Rashid	2012	0.72	0.46	0.28	0.85
Richie	1993	0.82	0.48	0.31	0.90
Seo	2007	0.98	0.04	0.33	0.87
Shahab	2013	0.98	0.19	0.13	0.98
Tauro	2009	1.00	0.38	0.40	1
Wymenga	2000	0.95	0.16	0.44	0.82

Three studies included in the meta-analysis collected stage and grade data for prostate cancer cases; however, none of these studies reported data for clinically significant prostate cancer diagnoses at a PSA cut-off of ≥ 4 ng/mL. Chang et al. [18] did not report the accuracy of PSA but showed a statistically significant difference in free to total PSA ratio for a Gleason Score of seven or more compared to Gleason Score of six or lower (11.69 ± 0.98 vs 16.47 ± 2.25, *p* = 0.029). Richie et al. [29] did not report the Gleason Score data collected but found higher PSA levels and increasing age were associated with a higher risk of metastatic prostate cancer. Shahab et al. [31] identified a PSA cut-off of 6.95 ng/mL for differentiating moderate versus high Gleason Score (which was not defined).

## Discussion

### Summary of findings

Published studies assessing the diagnostic accuracy of PSA in symptomatic patients reported high sensitivity and low specificity for the detection of prostate cancer. Eighteen of the included studies were undertaken in hospital urology outpatient populations, with one study focused on a symptomatic cohort within a population screening study. Importantly, there were no studies assessing the performance of PSA in a primary care population. Insufficient data was available to assess the diagnostic accuracy of PSA for clinically significant prostate cancer. Furthermore, all included studies had a high risk of bias in at least one QUADAS domain.

### Comparison to existing literature

Harvey et al. [10] published a systematic review of the diagnostic accuracy of PSA for prostate cancer in European populations, focused on studies published between 1998 and 2008. Individual study level data from 10 included papers was reported, though without estimating a combined level of accuracy. They considered the accuracy of PSA for all prostate cancer types overall and showed a range of accuracy estimates similar to this study. Over half of the studies included in this review were published since the review by Harvey et al. A review of clinical features of prostate cancer in primary care by Young and colleagues [34] in 2015 identified one study from 1989 of 287 patients referred from primary care with bladder outlet obstruction, of whom 211 had a PSA test. High levels of sensitivity (89.5%) and specificity (90%) were reported, but Young and colleagues considered the true level of accuracy was likely to be lower given few patients with a normal PSA level had the reference test for prostate cancer.

### Strengths and weaknesses

This study benefited from a rigorous, focused, methodological approach in conducting the review. All clinical settings were eligible, ensuring we found as many relevant studies as possible. Most included studies employed PSA in a similar manner, using similar indications and diagnostic thresholds, allowing for cross-study comparisons.

The evidence for the association between lower urinary tract symptoms and prostate cancer, particularly clinically significant prostate cancer, is equivocal. A number of secondary care studies suggest that symptoms do not discriminate well between prostate cancer and benign prostatic hypertrophy [35, 36]. This assumption is largely untested in primary care populations and contrasts with studies showing that the majority of patients diagnosed with prostate cancer present to their GP with LUTS prior to diagnosis [37–40]. This controversy also means that LUTS and other relevant symptoms may not be reported or be the focus of some potentially relevant studies of PSA for prostate cancer and may have limited the sensitivity of the search strategy employed. However, key papers were picked up by the database searches and the majority of PSA studies will likely be focused on screening in asymptomatic populations.

All included studies employed TRUS biopsy as a reference test, with some also including pathological data obtained from urological procedures on the prostate. TRUS biopsy is recognised as having poor sensitivity as a diagnostic test [41], owing to the inability to visualise lesions within the prostate resulting in a random sampling of the gland, and thus misclassification bias. Reporting of histological classification of prostate cancers was only included in three studies, and each presented this data differently. Insufficient data was available to determine a relationship between PSA and clinically significant prostate cancer, which is a crucial consideration for the optimal use of PSA for prostate cancer detection. Most included studies only performed the reference test on patients with a raised PSA or abnormal prostate examination, introducing partial verification bias. Therefore, the true sensitivity of PSA in symptomatic patients is unknown and likely to be lower than reported.

### Implications for research and practice

PSA is a commonly used test to assess for the presence of prostate cancer, mostly in a primary care setting, and is recommended as part of the assessment of patients with LUTS in national guidelines [42–44]. The lack of primary care evidence for the use of PSA to detect prostate cancer is known and is not the only condition for which secondary care evidence has been applied to primary care guidance [45]. Even so, this is a major gap in knowledge, as spectrum bias means that secondary care data (or screening data) do not translate to primary care. High-quality studies in primary care populations are needed to fill this gap, and future studies should report not just on prostate cancer per se but on clinically significant cancer as well. The introduction of more accurate diagnostic tests for prostate cancer, including multiparametric magnetic resonance imaging [41], increases the need for better understanding of the role of PSA in the early detection of symptomatic prostate cancer. PSA performance could also be enhanced by incorporating additional relevant clinical data in multivariable risk models [46], although only one has been validated in primary care [47].

Primary care clinicians are generally aware of the limitations of PSA testing [48], and clinical guidelines encourage a balanced discussion with patients of the potential benefits and harms of relying on PSA to detect prostate cancer [3, 49]. The findings of this review suggest this is a pragmatic approach in providing care to patients with LUTS. False-positive PSA results can also occur from non-cancer conditions affecting the prostate such as benign prostatic hypertrophy or prostatitis, further limiting the clinical utility of the test for prostate cancer detection. Alternative tests to PSA have been extensively researched [50, 51], and some show promise of improving the level of confidence in detecting prostate cancer, though none has entered primary care practice as yet.

## Conclusions

Published evidence from almost entirely secondary care based studies suggests that PSA has high sensitivity and low specificity for the diagnosis of prostate cancer in symptomatic patients. Published studies suffer from a number of biases, which probably overestimate the accuracy of PSA, and there were no included studies assessing the accuracy of PSA in a primary care population. The utility of PSA for the diagnosis of clinically significant prostate cancer in primary care remains unclear and needs urgent study. A major focus of such a study would be to identify patients with clinically significant cancer, warranting radical treatments, whilst avoiding exacerbating the issue of overdiagnosis of clinically insignificant prostate cancer.

## Supplementary Information

**Additional file 1.** Database search strategy.

**Additional file 2.** PRISMA 2020 Checklist.

**Additional file 3.** Supplementary figure 1—Fagan plot of included studies using PSA cut-off of 4ng/mL.

## Data Availability

All data were extracted from published research articles. The study protocol is available on PROSPERO and database search strategy is attached as an additional file.

## Abbreviations

AUC: Area under the curve
BPH: Benign prostatic hypertrophy
CI: Confidence interval
GP: General practitioner
HoLEP: Holmium laser enucleation of the prostate
HSROC: Hierarchical summary receiver operator curve
IPSS: International prostate symptom score
LUTS: Lower urinary tract symptoms
mpMRI: Multiparametric magnetic resonance imaging
MRI: Magnetic resonance imaging
ng/mL: Nanograms per millilitre
PCa: Prostate cancer
PSA: Prostate-specific antigen
SIGN: Scottish intercollegiate guidelines network
TNM: Tumour-node-metastasis
TRUS: Transrectal ultrasound-guided biopsy
TURP: Transurethral resection of the prostate
