# Supplementary material for: Systematic review and meta-analysis of the diagnostic accuracy of prostate-specific antigen (PSA) for the detection of prostate cancer in symptomatic patients
Source: BMC Med. 2022 Feb 7;20:54. doi: 10.1186/s12916-021-02230-y (PMC8819971; doi:10.1186/s12916-021-02230-y)
Supplement: Supplementary file 1 — Additional file 1. Database search strategy. [file 12916_2021_2230_MOESM1_ESM.docx]

Database search strategies

Medline

1. (prostat* adj3 (cancer* or carcinoma* or malignan* or tumo?r* or neoplas* or adeno*)).tw.
2. Exp Prostatic neoplasms/
3. Exp Prostatic Intraepithelial Neoplasia/
4. Exp prostate/
5. OR/1-4
6. exp "Sensitivity and Specificity"/
7. sensitivity.tw.
8. specificity.tw.
9. ((pre-test or pretest) adj probability).tw.
10. post-test probability.tw.
11. predictive value$.tw.
12. likelihood ratio$.tw.
13. diagnos*.tw
14. accura*.tw
15. OR/6-14
16. “prostate specific antigen”.mp
17. PSA.mp
18. OR/16-17
19. 5 AND 15 AND 18 Hits = 15,673 (05/02/21)
20. LUTS.tw
21. “lower urinary tract symptoms”.tw
22. 20 or 21
23. 19 and 22 Hits = 234 (05/02/21)

EMBASE

1. (prostat* adj3 (cancer* or carcinoma* or malignan* or tumo?r* or neoplas* or adeno*)).tw.
2. Exp prostate/
3. 1 or 2
4. “prostate specific antigen”
5. PSA
6. 4 or 5
7. Sensitive:.tw.
8. Diagnostic accuracy.sh.
9. Diagnostic.tw.
10. 7 or 8 or 9
11. 3 and 6 and 10 Hits = 13429 (05/02/21)
12. LUTS.tw.
13. “lower urinary tract symptoms”.tw.
14. 12 or 13
15. 11 and 15 Hits = 153 (05/02/21)

CENTRAL

#1 MeSH descriptor: [Prostate] explode all trees

#2 MeSH descriptor: [Prostatic neoplasms] explode all trees

#3 MeSH descriptor: [Prostatic Intraepithelial Neoplasia] explode all trees

#4 Prostat*(cancer or neoplasm* or carcin* or tumour* or tumor* or malignan* or neoplasia or adenocarcinoma*): ti,ab,kw

#5 #1 or #2 or #3 or #4

#6 “prostate specific antigen”:ti,ab,kw

#7 (PSA) :ti,ab,kw

#8 #6 AND #7

#9 MeSH descriptor: [Diagnosis] explode all trees

#10 #5 AND #8 AND #9 Hits = 1418 (05/02/21)

#11 (LUTS):ti,ab,kw

#12 (“lower urinary tract symptoms”):ti,ab,kw

#13 #11 OR #12

#14 #10 AND #13 Hits = 36 (05/02/21)

Web of science

#1 TS = (Prostate)

#2 TS = (cancer OR malignancy OR neoplas$ OR tumour OR adenocarcinoma)

#3 #1 AND #2

#4 TS = (PSA)

#5 TS = “prostate specific antigen”

#6 #4 OR #5

#7 TS=(diagnos* or accura* or sensitivit* or specificit* or likelihood or "positive predictive value" or PPV or "negative predictive value" or PPV or precision)

#8 #3 AND #6 AND #7 Hits = 15,041 (05/02/21)

#9 TS = LUTS

#10 TS = “lower urinary tract symptoms”

#11 #9 OR #10

#12 #8 AND #11 Hits = 208 (05/02/21)

Full search Total hits = 45,561

+ LUTS search = 631
