# Supplementary figures and images for: Systematic review and meta-analysis of the diagnostic accuracy of prostate-specific antigen (PSA) for the detection of prostate cancer in symptomatic patients
Source: BMC Med. 2022 Feb 7;20:54. doi: 10.1186/s12916-021-02230-y (PMC8819971; doi:10.1186/s12916-021-02230-y)

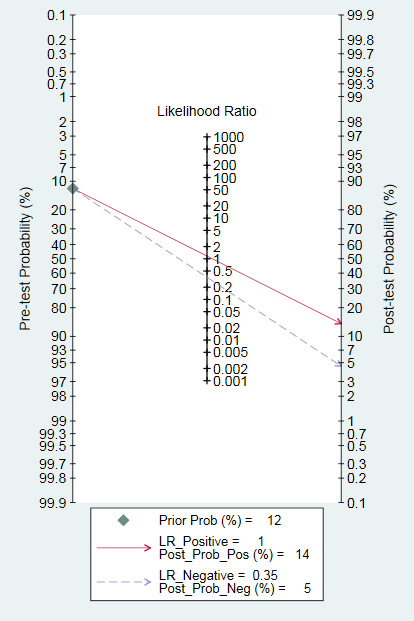

Supplement: Supplementary file 3 — Additional file 3. Supplementary figure 1—Fagan plot of included studies using PSA cut-off of 4ng/mL. [file 12916_2021_2230_MOESM3_ESM.png]
